# Differences in the risk of cervical cancer and human papillomavirus infection by education level

**DOI:** 10.1038/sj.bjc.6605224

**Published:** 2009-08-04

**Authors:** S Franceschi, M Plummer, G Clifford, S de Sanjose, X Bosch, R Herrero, N Muñoz, S Vaccarella

**Affiliations:** 1 International Agency for Research on Cancer, 150 cours Albert Thomas, 69372 Lyon cedex 08, France; 2 Servei d'Epidemiologia i Registre del Cancer, Institut Català d'Oncologia, Hospital Duran i Reynals, Av. Gran Via, s/n. Km 2.7, 08907 L'Hospitalet de Llobregat, Barcelona, Spain; 3 Proyecto Epidemiológico Guanacaste, Fundación INCIENSA, Torre La Sabana, 300 Oeste del ICE, Piso 7, Sabana Norte, San José, Costa Rica; 4 Instituto Nacional de Cancerología, Calle I No. 9-85, Bogota, Colombia

**Keywords:** cervical cancer, human papillomavirus, education, age at first sexual intercourse, age at first pregnancy

## Abstract

**Background:**

Cervical cancer risk is associated with low education even in an unscreened population, but it is not clear whether human papillomavirus (HPV) infection follows the same pattern.

**Methods:**

Two large multicentric studies (case–control studies of cervical cancer and HPV prevalence survey) including nearly 20 000 women. GP5+/GP6+ PCR was used to detect HPV.

**Results:**

Education level was consistently associated with cervical cancer risk (odds ratio (OR) for 0 and >5 years *vs* 1–5 years=1.50, 95% confidence interval (CI): 1.25–1.80 and 0.69, 95% CI: 0.57–0.82, respectively, *P* for trend <0.0001). In contrast, no association emerged between education level and HPV infection in either of the two IARC studies. A majority of the women studied had never had a Pap smear. The association between low education level and cervical cancer was most strongly attenuated by adjustment for age at first sexual intercourse and first pregnancy. Parity and screening history (but not lifetime number of sexual partners, husband's extramarital sexual relationships, and smoking) also seemed to be important confounding factors.

**Conclusion:**

The excess of cervical cancer found in women with a low socio-economic status seems, therefore, not to be explained by a concomitant excess of HPV prevalence, but rather by early events in a woman's sexually active life that may modify the cancer-causing potential of HPV infection.

Cervical cancer risk is associated with a low socio-economic status (SES), as defined by education or income levels (Parikh *et al*, 2003). The reasons for the association are not fully understood but are thought to include a higher prevalence of cervical cancer risk factors, such as inadequate cervical cancer screening (Khan *et al*, 2005), high parity (International Collaboration of Epidemiological Studies of Cervical Cancer, 2006), and possibly high-risk sexual behaviour (International Collaboration of Epidemiological Studies of Cervical Cancer, 2009; Louie *et al*, 2009) among women with a low SES (de Sanjose *et al*, 1997).

It is now believed that the majority of cervical cancer is preceded by a long-term infection with high-risk types of the human papillomavirus (HPV), a sexually transmitted infection (Schiffman *et al*, 2007). High prevalence of HPV in women with a low SES has been shown in a few studies (Hildesheim *et al*, 1993; Lazcano-Ponce *et al*, 2001), but has not been confirmed in others (Deacon *et al*, 2000; Shapiro *et al*, 2003; Khan *et al*, 2005; Banura *et al*, 2008; Hibbitts *et al*, 2008).

We took advantage of two large series of studies coordinated by the International Agency for Research on Cancer (IARC), that is, the IARC Multicentric Case–Control Study (Munoz *et al*, 2002) and the IARC HPV Prevalence Surveys (Franceschi *et al*, 2006), to evaluate the relationship between education level and risk of cervical cancer and HPV infection.

## Materials and methods

### The IARC multicentric case–control study

**Figure 1:**
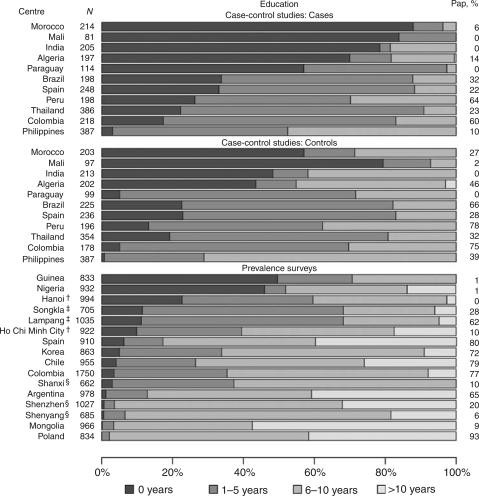
Distribution of women with and without cervical cancer in the International Agency for Research on Cancer case–control studies and human papillomavirus prevalence surveys, according to education level, Pap smear history^*^, and study area. ^*^In case–control studies, Pap smears taken 12 months before enrolment are excluded. ^†^Study areas in Vietnam. ^‡^Study areas in Thailand. ^§^Study areas in China.

Methods have been described earlier for the 11 individual studies carried out between 1985 and 1999 that have been included in this paper (Munoz *et al*, 1992; Eluf-Neto *et al*, 1994; Chaouki *et al*, 1998; Chichareon *et al*, 1998; Ngelangel *et al*, 1998; Rolon *et al*, 2000; Santos *et al*, 2001; Bayo *et al*, 2002; Franceschi *et al*, 2003; Hammouda *et al*, 2005). Briefly, eligible cases were residents in predefined study areas, or women attending reference hospitals with incident, histologically confirmed invasive cervical cancer. A total of 2446 cases were identified, including 140 adeno- or adenosquamous invasive cervical carcinomas from six study areas (Eluf-Neto *et al*, 1994; Chaouki *et al*, 1998; Chichareon *et al*, 1998; Ngelangel *et al*, 1998; Rolon *et al*, 2000; Santos *et al*, 2001). Control women were population based in the Spanish and Colombian studies (Munoz *et al*, 1992) and hospital or clinic based in other study areas. They were frequency matched by 5-year age group and did not include women admitted to hospital for cancers of the anogenital tract, breast and colon, smoking-related diseases (Munoz *et al*, 1992), or sexually transmitted infections (Hammouda *et al*, 2005). A total of 2390 control women were included between 1985 and 1999 (Figure 1).

### The IARC HPV prevalence surveys

Sampling methods of participating women have already been described for the 16 individual studies carried out between 1993 and 2006 that are included in this paper (Molano *et al*, 2002; Anh *et al*, 2003; de Sanjose *et al*, 2003; Matos *et al*, 2003; Shin *et al*, 2003; Sukvirach *et al*, 2003; Ferreccio *et al*, 2004; Thomas *et al*, 2004; Dai *et al*, 2006; Li *et al*, 2006; Wu *et al*, 2007; Bardin *et al*, 2008; Dondog *et al*, 2008; Keita *et al*, 2009). Briefly, in each area attempts were made to obtain a population-based sample that included at least 100 women in each 5-year age group between 15 or 18 years, and 55 years or older. Participation ranged from 48% to over 90% and, overall, 15 051 women were included. On account of the need to undergo a gynaecological examination, prevalence surveys were mainly restricted to women who reported to have had sexual intercourse and, in some areas, to married women. Few single women (6% of all study women) and only 90 virgin women were, therefore, included.

The questionnaires used in both case–control studies and prevalence surveys included a question with regard to the years of full-time education and information on lifetime number of sexual partners, age at first sexual intercourse, husband's extramarital sexual relationships, use of oral contraceptives and condom, parity, age at first pregnancy, smoking, and history of Pap smear. Among cases, any Pap smear taken less that 1 year before cancer diagnosis was excluded as they were considered ‘diagnostic smears’.

All women in both studies signed informed consent forms according to the recommendations of the IARC and local ethical review committees, which approved the studies.

### HPV detection

HPV testing was performed in the Department of Pathology at the Vrije University Medical Center, Amsterdam, the Netherlands, as described in individual study publications. The overall presence of HPV DNA was determined by performing a general primer GP5+/6+-mediated PCR (Jacobs *et al*, 2000). HPV positivity was assessed by hybridisation of PCR products in an enzyme immunoassay using two HPV oligoprobe cocktails that, together, detect the following HPV types: HPV6, 11, 16, 18, 26, 31, 33, 34, 35, 39, 40, 42, 43, 44, 45, 51, 52, 53, 54, 55, 56, 57, 58, 59, 61, 66, 68, 70, 72, 73, 82 (IS39 and MM4 subtypes), 83 (equivalent to MM7), 84 (equivalent to MM8), and CP6108. Subsequent HPV typing was performed by reverse-line blot hybridisation of PCR products, as described earlier (Jacobs *et al*, 1995; van den Brule *et al*, 2002).

### Statistical analysis

Education level was classified into four groups (0, 1–5, 6–10; >10 years). Owing to small numbers, the last two groups were merged in the case–control studies. Regularised logistic regression (Gelman *et al*, 2008) was used to calculate odds ratios (ORs) and 95% confidence intervals (CIs) by education level for cervical cancer in the case–control studies and for HPV infection among control women only in case–control studies and among the general female population in prevalence surveys. The reference category for education was set to the most common category (i.e., 1–5 years in case–control studies and 6–10 years in prevalence surveys).

Tests for trend were computed using three or four categories of education level as continuous variables. All analyses were adjusted for age (in 5-year groups) and study area. Adjustment was also made for lifetime number of sexual partners (0–1, 2, ⩾3), age at first sexual intercourse (<17, 17–20, ⩾21 years), husband's extramarital sexual relationships (no, yes, or uncertain), number of full-term pregnancies (0, 1–2, 3–4, 5–6, ⩾7), age at first pregnancy (<18, 18–20, ⩾21 years), use of hormonal contraceptives and condom (never, ever), smoking (never, former, ever), and history of Pap smear (never, ever), as reported.

Heterogeneity of ORs between study areas was tested by fitting separate models to each area and then comparing the observed with the expected dispersion of estimates around the pooled mean using a *χ*^2^ statistic. For purposes of assessing heterogeneity, study areas with five or fewer individuals in a given category of education were not considered, as they could not provide OR estimates. All statistical tests were two-sided.

Results in the text are presented as ORs with conventional CIs. Where results are presented in the form of plots, floating absolute risks (Plummer, 2004) were used to represent the dose–response relationship in a way independent of the choice of reference category. ORs are represented by squares and their corresponding floating CIs by horizontal lines. The position of the square indicates the point estimate of the OR, and the area of the square is inversely proportional to the square of the floating standard error on the log scale, thus providing an indication of the amount of statistical information available.

## Results

Figure 1 shows the number of women included in each study and the broad variations in education level across study areas. Among cervical cancer cases, 82% (range: 53–100%) reported 5 years of education or less. The percentage of women who reported 5 years of education or less was 66% (range 29–93%) among control women in the IARC Multicentric Case–Control Study and 34% (range: 2–71%) among the general female population in the IARC HPV Prevalence Surveys (Figure 1). Large variations were also found in the proportion of women who reported to have had a Pap smear, averaging 23% (range: 0–64%) and 37% (range: 0–78%) among cervical cancer cases and control women, respectively, in case–control studies, and 42% (range: 0–93%) among women in prevalence surveys (Figure 1).

**Figure 2:**
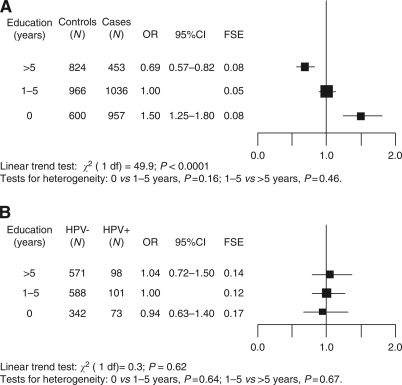
Odds ratios (OR)^†^ and corresponding 95% confidence intervals (CI) for (**A**) cervical cancer risk and (**B**) human papillomavirus (HPV) positivity among control women only. The International Agency for Research on Cancer Multicentric Case–Control Study. FSE=floating standard error, HPV=human papillomavirus. ^†^Adjusted for age, study area, lifetime number of sexual partners, age at first sexual intercourse, husbands' extramarital sexual relationships, parity, age at first pregnancy, oral contraceptive use, and history of Pap smear.

Education level was associated with cervical cancer risk (OR for 0 and >5 years *vs* 1–5 years=1.50, 95% CI: 1.25–1.80; and 0.69, 95% CI: 0.57–0.82, respectively, *P* for trend <0.0001) in case–control studies (Figure 2A). The association was similar in all study areas and no statistically significant heterogeneity emerged. When the association between education level and risk of adeno- or adenosquamous carcinoma was evaluated separately, findings were similar to those for all cervical cancers (OR for 0 and >5 years *vs* 1–5 years=1.80, 95% CI: 1.11–2.91; and 0.76, 95% CI: 0.47–1.24, respectively, data not shown). In contrast, education level was not associated with HPV positivity among control women (OR for 0 and >5 years *vs* 1–5 years=1.04, 95% CI: 0.63–1.40; and 0.94, 95% CI: 0.63–1.40) (Figure 2B).

**Figure 3:**
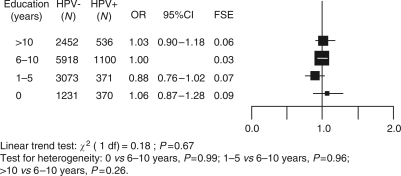
Odds ratios (OR)^†^ and corresponding 95% confidence intervals (CI) for human papillomavirus (HPV) positivity by education level. The International Agency for Research on Cancer HPV Prevalence Surveys. FSE=floating standard error. ^†^Adjusted for age, study area, lifetime number of sexual partners, age at first sexual intercourse, husbands' extramarital sexual relationships and history of Pap smear.

In agreement with the findings among control women in the case–control studies, no association between education level and HPV positivity was found among the larger number of women included in the prevalence surveys (OR for >10, 1–5, and 0 years *vs* 6–10 years=1.03, 95% CI: 0.90–1.18; 0.88, 95% CI: 0.76–1.02; and 1.06, 95% CI: 0.87–1.28, respectively, *P* for trend=0.67) (Figure 3). Although some differences emerged across study areas with some non-statistically significant associations in either direction, no significant heterogeneity was found between study areas with respect to HPV infection and education level.

The influence of adjusting for different confounding variables on the association between cervical cancer risk and education (dichotomised as ⩽5 and >5 years) in the case–control studies is shown in Table 1. The age- and study area-adjusted OR (2.64, 95% CI: 2.27–3.06) was most strongly attenuated by adjustment for age at first sexual intercourse (OR=2.03, 95% CI: 1.73–2.37) and age at first pregnancy (OR=2.13, 95% CI: 1.80–2.52). Adjustment for parity, use of oral contraceptives, and history of Pap smear also somewhat diminished the OR by education level, whereas adjustment for lifetime number of sexual partners, husband's extramarital sexual relationships, condom use, and tobacco smoking did not. The fully adjusted OR for ⩽5 *vs* >5 years of education was 1.41 (95% CI: 1.11–1.79), that is, 47% lower than the OR adjusted for age and study area only.

## Discussion

Education level was inversely and consistently associated with cervical cancer risk, but not with HPV prevalence, in a broad range of world populations included in the IARC studies. The lack of excess of HPV positivity in low-education women was consistently found among control women in case–control studies (i.e., in the same population in which the association between low-education level and cervical excess risk emerged), as well as in the larger number of women included in the HPV prevalence surveys that were carried out in part in different countries than the case–control studies.

Obviously, education level cannot be considered as a well-defined exposure, but as a marker for a combination of characteristics that predominate among women with a low SES. The aim of our study was not, therefore, to rule out bias and confounding, but rather to evaluate the principal risk factors responsible for any socio-economic gradient.

In screened populations, education level is most likely to be a surrogate of inadequate screening (Khan *et al*, 2005), but a socio-economic gradient in cervical cancer risk was reported long before screening programmes were introduced (Jones *et al*, 1958) and is still seen in countries where little screening activity exists (Franceschi *et al*, 2003; Parikh *et al*, 2003). Such was the case in our combined analysis; although in some study areas, a large fraction of women who reported to have had at least one Pap smear taken in their lifetime and screening history did have an influence on the association with education level, nowhere did broad-coverage and high-quality screening programmes exist at the time our studies took place (Konno *et al*, 2008; Murillo *et al*, 2008).

A substantial fraction of the influence of socio-economic gradient on cervical cancer risk in our study was explained by the early age of first sexual intercourse and first pregnancy (Louie *et al*, 2009), as well as by high parity, whereas lifetime number of sexual partners and husband's extramarital sexual relationships did not seem to be important confounding factors. Similarly, adjustment for the presence of HPV infection among cervical cancer cases and controls or restriction to HPV-positive women, as carried out in some earlier reports from the IARC case–control study (Munoz *et al*, 2002), left the association with low education level unchanged (data not shown).

The weaknesses of this study include the limitation in the completeness and quality of information available on SES, which led us to use education level only as a proxy of SES, and on possible confounding factors, notably sexual behaviour and screening history. Residual confounding, therefore, probably still inflates the association between cervical cancer risk and education in our report. This problem does not eclipse, however, the clear difference we found between cervical cancer and HPV positivity with respect to SES. Additional information on SES (e.g., income, ownership of the house, and number of appliances and facilities at home) and husband's sexual behaviour (e.g., sexual intercourses with prostitutes) (de Sanjose *et al*, 1997) was included in some studies that are a part of this report. These variables, however, were missing for a substantial proportion of women and, in the case of SES indicators, were highly correlated with education level. The strengths of this study are the large number of women included, the performance of high-quality HPV testing, and the relatively modest impact of cervical cancer screening in the majority of populations in which IARC studies have been performed that allowed other correlates of low education level other than screening to emerge more clearly.

Our findings imply that higher HPV prevalence and lifetime number of sexual partners do not explain the excess of cervical cancer in women with low SES. Conversely, early events that can modify the carcinogenic potential of HPV infection, such as early age of first intercourse (a possible proxy of longer duration of uncleared HPV infection (International Collaboration of Epidemiological Studies of Cervical Cancer, 2009)) and early and multiple pregnancies (International Collaboration of Epidemiological Studies of Cervical Cancer, 2006) account for a large part of the SES gradient of cervical cancer, at least in inadequately screened populations.

## Figures and Tables

**Table 1:** Effect of adjustment by potential confounding factors on the odds ratio (OR) of cervical cancer by education level

**Adjustment variables**	**OR (95% CI) ⩽5 *vs* >5 years of education**
Age+study area	2.64 (2.27–3.06)
	
*As above*+	
Lifetime number of sexual partners	2.55 (2.19–2.97)
Age at first intercourse	2.03 (1.73–2.37)
Husband's extramarital sexual relationships	2.62 (2.21–3.11)
Parity	2.22 (1.90–2.59)
Age at first pregnancy	2.13 (1.80–2.52)
Oral contraceptive use	2.40 (2.02–2.84)
Condom use	2.61 (2.25–3.03)
Smoking	2.65 (2.28–3.08)
History of Pap smear	2.25 (1.92–2.62)
All the above (except condom use and smoking)	1.41 (1.11–1.79)

CI=confidence interval.

## Appendix

In addition to the aforementioned, the members of the IARC Multicentric Case-Control Study Group are (in alphabetical order by country): Algeria (D Hammouda); Brazil (J Eluf-Neto); Colombia (N Aristizabal, LA Tafur); India (T Rajkumar); Mali (S Bayo); Morocco (N Chaouki, B El Gueddari); the Netherlands (CJLM Meijer, PJF Snijders, AJC van den Brule, JMM Walboomers); Paraguay (PA Rolón); Peru (E Caceres, C Santos); the Philippines (C Ngelangel); Spain (N Ascunce, X Castellsagué, M Gili, LC González, I Izarzugaza, C Navarro, M Santamaria); Thailand (S Chichareon); and the United States (KV Shah).

In addition to the aforementioned, the members of the IARC Human Papillomavirus Prevalence Surveys Study Group are (in alphabetical order by country): Argentina (D Loria, E Matos); Chile (C Ferreccio, A Luzoro, JM Ojeda, R Prado); China (M Dai, N Li, YL Qiao, RF Wu); Colombia (M Molano, H Posso); France (A Arslan); Guinea (N Keita); Korea (D-H Lee, H R Shin); Mongolia (B Dondog, M Pawlita); Nigeria (A Omigbodun, JO Thomas); Poland (A Bardin, W Zatonski); Thailand (S Sukvirach, S Tunsakul); The Netherlands (CJLM Meijer, PJF Snijders); the United States (J Smith); and Vietnam (PTH Anh, NT Hieu).
